# Intervention of ginseng-derived macromolecular drugs in Alzheimer’s disease: exploring mechanisms and assessing potential

**DOI:** 10.3389/fnagi.2026.1752446

**Published:** 2026-03-24

**Authors:** Weiqiao Liu, Yunfan Bai, Wenyi Qu, Xiaoying Zhang, Shuo Zhou, Haoming Luo, Ming Zhu, Dongyang Li, Xiaoxue Fang

**Affiliations:** 1 College of Pharmacy, Changchun University of Chinese Medicine, Changchun, China; 2 The First Hospital of Jilin University, Changchun, China

**Keywords:** Alzheimer’s disease, amyloid-β, ginseng, neuroprotection, polysaccharides

## Abstract

Alzheimer’s disease (AD) is a prevalent neurodegenerative disorder for which effective treatments remain elusive. This review aims to explore the roles, mechanisms, and therapeutic potential of three principal ginseng components, including ginseng polysaccharides (GPS), ginseng proteins (GP), and ginseng glycoproteins (GGP), in the prevention and management of AD. We systematically reviewed recent literature related to these components in AD research. By analyzing evidence from cellular experiments, animal models, and preliminary clinical studies, we evaluated their effects on core pathological processes. These ginseng-derived compounds exert neuroprotective effects via multiple pathways. Specifically, they inhibit the aggregation of amyloid-β (Aβ) and reduce the hyperphosphorylation of tau protein. Furthermore, they demonstrate significant anti-neuroinflammatory and antioxidant activities, which protect neurons from damage and enhance cognitive functions, including memory and learning. The efficacy of these components has been consistently demonstrated across various AD experimental models. In conclusion, GPS, GP, and GGP exhibit promise as multitarget therapeutic agents against AD, underscoring a potential pathway for developing novel natural product-based treatments. Although current preclinical results are promising, further rigorous clinical trials are necessary to validate their efficacy and safety in humans. Therapeutic strategies targeting these components may therefore offer new hope for AD patients.

## 1 Introduction

AD a chronic, degenerative central nervous system disorder and the leading cause of dementia worldwide. It is primarily characterized by a range of neuropsychiatric symptoms. Epidemiological studies estimated that approximately 57 million people were living with dementia globally in 2021, a figure projected to reach 139 million by 2050. The high prevalence of AD imposes a substantial burden, severely compromising patients’ quality of life. Currently, nearly 10 million new cases are reported each year, translating to one new diagnosis every 3.2 s (Alzheimer’s Disease International, 2021; World Health Organization, 2021). The hallmark pathological features of AD include the abnormal aggregation of Aβ into extracellular senile plaques and the hyperphosphorylation of tau protein, which forms neurofibrillary tangles (NFTs) (Chen et al., 2016; Davtyan et al., 2019; Howlett and Richardson, 2009). These pathological changes collectively lead to neuronal loss and synaptic dysfunction. Clinically, AD manifests as a progressive neuropsychiatric disorder, with core symptoms including short-term memory loss, aphasia, disorientation, and executive dysfunction, along with potential symptoms such as auditory hallucinations, delusions, paranoia, personality changes, and a declining ability to perform familiar activities (Collette et al., 1999; Doody et al., 1995; Lerner et al., 1994). In advanced stages, patients often lose basic self-care capabilities. Despite decades of research, current clinical pharmacotherapy for AD remains limited. The mainstay treatments include cholinesterase inhibitors (e.g., donepezil) and N-methyl-D-aspartate (NMDA) receptor antagonists (e.g., memantine). However, these drugs offer only temporary symptomatic relief without altering the disease’s progression (Seow and Gauthier, 2007). Their cognitive benefits are modest, and they can be associated with significant side effects. Therefore, developing safe and effective treatment strategies represents a critical and urgent unmet medical need. To address these limitations, the paradigm of AD drug discovery is shifting toward multi-target directed ligands, such as dual inhibitors of AChE and BACE1 (Shrivastava et al., 2022). There is also a resurgent interest in natural bioactive molecules (e.g., baicalein and punicalagin) and traditional medicinal plants, which offer multifaceted neuroprotective benefits (Siddiqui et al., 2024a,b; Tripathi et al., 2024). Moreover, the advancement of precision neurology, particularly the use of neurofilament biomarkers, has further improved the monitoring of disease progression and therapeutic response (Sharma et al., 2024).

In this context, ginseng (*Panax ginseng* Meyer), a perennial herb of the Araliaceae family, remains a significant source of bioactive molecules. It has been used in East Asia for over two thousand years and is traditionally believed to possess potent cognition-boosting effects (Petkov et al., 2003). Modern research has identified a diverse profile of bioactive constituents in ginseng, including ginsenosides, polysaccharides, proteins, and volatile oils (Cui et al., 2016; Guo et al., 2021; Tao et al., 2025). These compounds exhibit broad pharmacological activities, such as neuroprotection, anti-inflammation, antioxidant, and antidiabetic effects, contributing to their disease preventive and therapeutic potential (Chen et al., 2022; El-Sheikh and Kamel, 2016; Jin et al., 2019; Naseri et al., 2022; Park et al., 2017; Zhai et al., 2022). Numerous studies have reported the beneficial effects of ginseng on cognitive functions relevant to AD (Hwang et al., 2025; Lee et al., 2023; She et al., 2024). While earlier research primarily focused on ginsenosides (e.g., Rg1 and Rb1) for AD treatment, recent investigations have expanded to include other bioactive components, notably GPS, GP, and GGP. Among these, GPS have demonstrated significant immunomodulatory (Jiang et al., 2023), antioxidant (Guo et al., 2023) and tumor microenvironment modulating activities (Li et al., 2021). GP encompass a range of functional proteins, RNA-like proteins, ribonucleases, saponin synthesis-related enzymes, chitinases, and xylanases (Ebadzadsahrai et al., 2018; Kumar et al., 2019; Talamantes et al., 2016). They are reported to mitigate AD pathology through multiple signaling pathways; for example, by upregulating mRNA and protein expression levels of Bcl-2/Bax, p-Akt/Akt, and p-PI3K/PI3K, thereby reducing Aβ_1_–_42_ and phosphorylated tau (p-tau) levels in the hippocampus (Li et al., 2016). GGP are glycoconjugates that integrate the properties of polysaccharides and proteins, constituting a significant class of bioactive macromolecules in traditional Chinese medicine (Hu et al., 2022). Furthermore, GGP show unique promise in improving learning and memory (Luo et al., 2018), modulating inflammatory responses (Lorz et al., 2020) and regulating immunity.

Unlike previous literature that primarily focuses on ginseng saponins, this review systematically outlines the roles of GPS, GP, and GGP for AD prevention and treatment by integrating multi-target evidence from cellular experiments and cross-species animal models. It also evaluates their potential for clinical translation and proposes specific recommendations for preclinical standardization, providing a comprehensive theoretical foundation for developing novel ginseng-based therapies for AD.

## 2 Hypothesis on the pathogenesis of AD

AD is a progressive neurodegenerative disorder, clinically evolving through preclinical AD, mild cognitive impairment, and AD dementia, and etiologically classified as familial or sporadic forms, with both exhibiting progressive decline in memory, judgment, and abstract thinking (Kalaria and Sepulveda-Falla, 2021). The established pathological hallmarks include Aβ deposits forming senile plaques and hyperphosphorylated tau protein forming NFTs (Kong et al., 2020). Furthermore, the severity of AD is closely linked to degeneration of the cholinergic system. For instance, selective loss of cholinergic neurons can induce symptoms such as memory impairment and cognitive confusion. As neuronal damage worsens, patients may develop more severe functional decline, including manifestations such as dysphagia (Lennol et al., 2025; Zhang et al., 2019). The pathogenesis of AD is explained by several interconnected hypotheses, principally centered on Aβ and tau pathology, while also involving neuroinflammation and oxidative stress, which collectively lead to synaptic loss and cholinergic neuronal degeneration (Li et al., 2023; Vassar et al., 1999). It is critical to note that AD onset is not driven by a single pathway, but rather by a complex network process initiated by core pathological proteins, amplified through neuroinflammation and oxidative stress, and modulated by genetic and metabolic risk factors. This section will systematically delineate the intrinsic connections among these hypotheses under a unified framework to provide integrated theoretical insights and potential targets for novel AD drug development. The various interconnected hypotheses regarding the pathogenesis of AD are summarized in Table 1.

**TABLE 1:** Summary of major Alzheimer’s disease hypotheses and their interconnected pathogenesis.

AD hypothesis	Primary pathological drivers/mechanisms	Interconnections with other hypotheses
Amyloid cascade	Overproduction, misfolding, and aggregation of Aβ peptides (Aβ_40_/Aβ_42_) into toxic oligomers and senile plaques.	Acts as an early trigger; directly induces neuroinflammation and oxidative stress; accelerates tau hyperphosphorylation.
Tau pathology	Abnormal hyperphosphorylation of tau protein, leading to microtubule destabilization and neurofibrillary tangles (NFTs).	Triggered by Aβ and oxidative stress; sequesters APP endosomes to promote further Aβ production in a feed-forward cycle.
Neuroinflammation	Chronic activation of microglia and astrocytes; excessive release of pro-inflammatory cytokines (e.g., IL-1β).	Initiated by Aβ/tau deposits; exacerbates Aβ aggregation and tau phosphorylation; closely linked to APOE4 dysfunction.
Oxidative stress and mitochondrial dysfunction	Overproduction of ROS/RNS; impaired respiratory chain complexes; decline in baseline mitochondrial function.	Catalyzed by metal ion dyshomeostasis; promotes Aβ aggregation and tau phosphorylation; activates neuroinflammation.
Metal ion dyshomeostasis	Age-related imbalances and accumulation of trace metals (Cu^2+^, Zn^2+^, Fe^2+^) in the brain.	Directly interacts with Aβ to accelerate amyloid deposition; catalyzes ROS generation, driving oxidative stress.
Cholinergic system damage	Degeneration of basal forebrain cholinergic neurons; decline in ACh levels and ChAT activity.	Exacerbated by Aβ/tau pathology, neuroinflammation, and oxidative stress, forming a vicious loop of cognitive decline.
Cholesterol deficiency and APOE	Downregulation of DHCR24; APOE4-mediated impairment of lipid transport and Aβ clearance.	Connects to multiple risk factors; promotes Aβ production/aggregation, induces apoptosis, and fosters neuroinflammation.
Other pathways	Glutamate excitotoxicity; impaired autophagic flux; insulin resistance.	Overactivation of receptors links to aberrant glial responses (neuroinflammation); autophagic failure prevents Aβ/tau clearance.

### 2.1 Aβ plaque deposition

**FIGURE 1:**
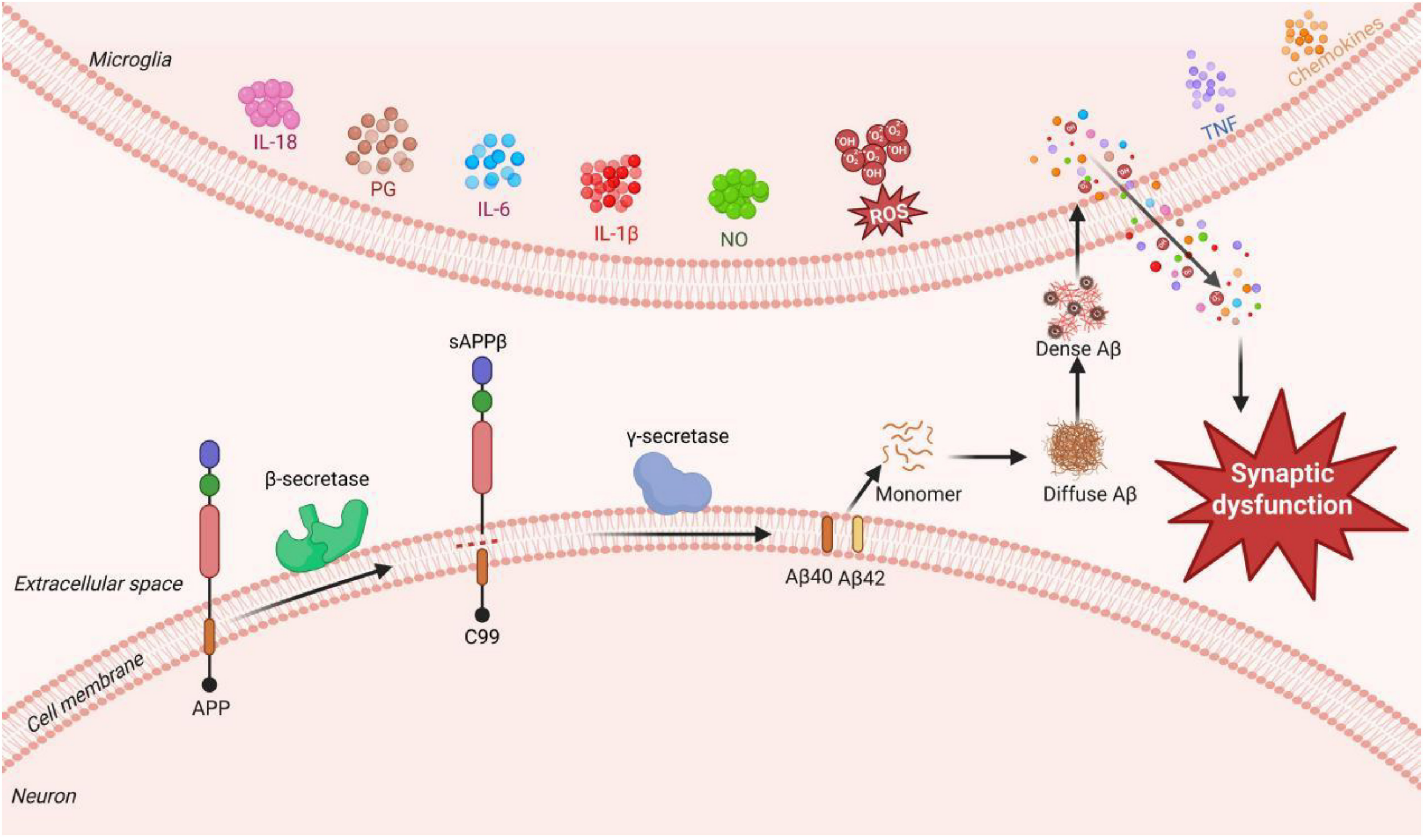
The amyloid cascade hypothesis in AD pathogenesis.

The amyloid cascade hypothesis, first proposed by Hardy and Allsop (1991), remains one of the most influential etiological theories of AD (Nalivaeva et al., 2020). It posits that the aberrant production, misfolding, aggregation, and deposition of Aβ peptides into senile plaques constitute the initiating event that triggers the neurodegenerative cascade. Aβ is generated through the sequential proteolytic cleavage of amyloid precursor protein (APP) by β- and γ-secretases (Salminen et al., 2009). Under physiological conditions, Aβ homeostasis is maintained by enzymatic degradation, mediated by zinc metalloproteases (e.g., neprilysin), endothelin-converting enzymes, and insulin-degrading enzyme (Yu et al., 2024), and by clearance mechanisms such as microglial phagocytosis (Wilcock et al., 2004). Pathologically, however, altered APP processing promotes the overproduction of Aβ peptides, particularly the Aβ_42_ (Crespo et al., 2014). Among these, Aβ_40_ and Aβ_42_ are the most prevalent in the brain and are critical to AD pathogenesis (Kametani and Hasegawa, 2018; Vigo-Pelfrey et al., 1993). An elevated Aβ_42_ level or an increased Aβ_42_/Aβ_40_ ratio drives the formation of amyloidogenic fibrils, whose accumulation into senile plaques exerts neurotoxicity, ultimately leading to neuronal death and neurodegenerative decline (Kametani and Hasegawa, 2018). Of particular significance, Aβ_42_ is highly amyloidogenic. Upon misfolding, it assembles through β-sheet formation and hydrophobic interactions into toxic oligomers, protofibrils, mature fibrils, and finally, senile plaques (Crespo et al., 2014). This aggregation process not only directly impairs neuronal function but also engages in crosstalk with other pathological pathways, such as neuroinflammation and oxidative stress, forming a vicious cycle that accelerates disease progression. Moreover, soluble amyloid-β oligomers (AβOs) are recognized as critical mediators of synaptic dysfunction (Wilcox et al., 2011), AβOs can trigger reactive gliosis, leading to the release of toxic factors that exacerbate neuronal damage (Forloni and Balducci, 2018), as summarized in Figure 1.

Importantly, targeted clearance of Aβ by microglia has been shown to ameliorate behavioral deficits and neuropathology in AD mouse models (Lin et al., 2024), highlighting the therapeutic potential of modulating neuroimmune function. Although Aβ deposition is a recognized early hallmark of AD and has spurred numerous drug development campaigns, most clinical trials targeting Aβ or its pathways have failed over the past three decades (Giacobini and Gold, 2013). This collective lack of success implies that Aβ accumulation may serve as an early trigger in the disease cascade rather than the sole driver of late stage symptomatology. Consequently, ongoing research continues to refine this hypothesis, aiming to inform the development of next-generation AD therapeutics.

### 2.2 Abnormal post-translational modification of tau protein

**FIGURE 2:**
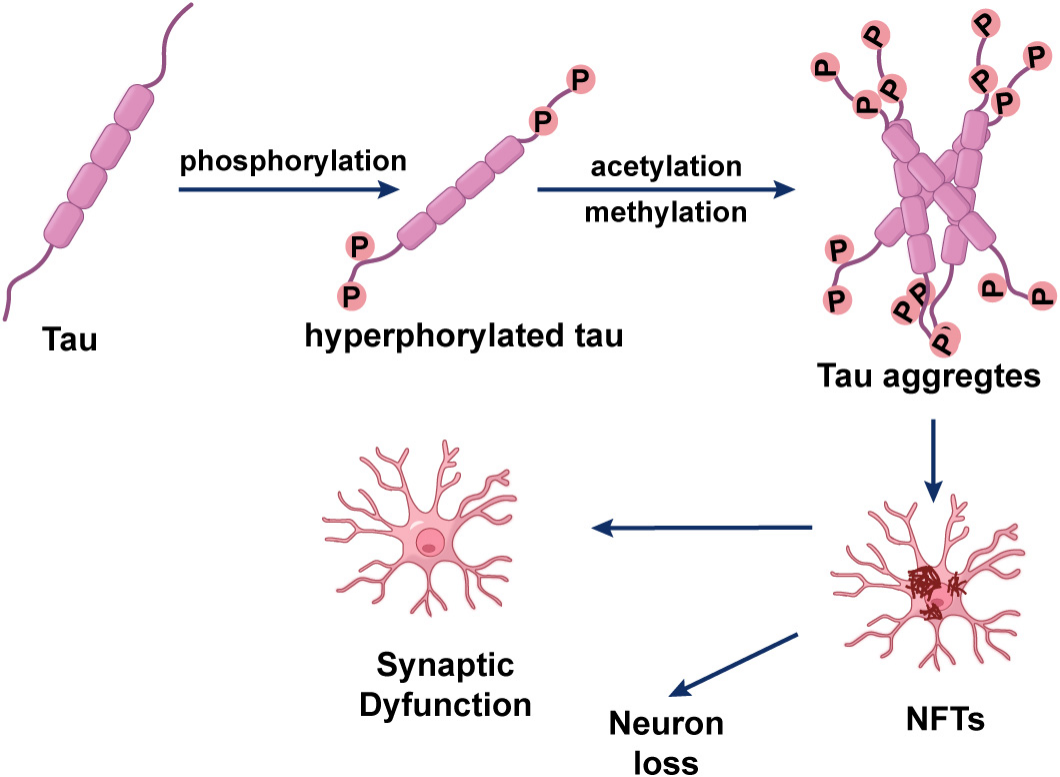
Tau protein hyperphosphorylation and neurofibrillary tangle formation.

Tau, a microtubule associated protein, is critical for maintaining neuronal microtubule stability (Cleveland et al., 1977). Under physiological conditions, tau binds to tubulin to promote the assembly and stability of the microtubule network. In AD, however, tau undergoes abnormal hyperphosphorylation. This charge-altering modification causes tau to detach from microtubules and self-aggregate, ultimately forming NFTs (Goedert et al., 1989; Kosik et al., 1986; Spires-Jones et al., 2009). NFT burden correlates positively with the severity of clinical symptoms, as these aggregates disrupt neuronal signaling and trigger cell death (Arnsten et al., 2021; Mohandas et al., 2009). Consequently, the cascade of tau hyperphosphorylation, aggregation, and deposition is recognized as a core pathological hallmark of AD (Arnsten et al., 2021; Maccioni et al., 2010). Within the AD pathological continuum, Aβ plaques are widely considered an initiating factor, while tau pathology is a central driver of synaptic loss and neuronal circuit failure. At a molecular level, Aβ pathology can trigger and exacerbate tau hyperphosphorylation, thereby promoting its dissociation from microtubules and subsequent misfolding into NFTs. This interplay is driven by several mechanisms, including the activation of specific kinases (e.g., GSK-3β), induction of neuroinflammation, impairment of proteasomal degradation of tau, and disruption of axonal transport (Blurton-Jones and Laferla, 2006). These two core pathologies thus engage in a feed-forward cycle that accelerates irreversible neurodegeneration. Beyond phosphorylation, additional post-translational modifications of tau, such as acetylation, glycosylation, and methylation, also regulate its aggregation, toxicity, and pathological progression (Mandelkow and Mandelkow, 2012). For example, Cohen et al. (2011) demonstrated that tau acetylation disrupts its binding to microtubules, thereby compromising microtubule function and cytoskeletal integrity. Furthermore, lysine methylation of tau has been shown to facilitate neurofibrillary lesion formation and promote neuronal apoptosis (Thomas et al., 2012). Notably, the toxicity of tau is not confined intracellularly. Pathological tau species can undergo trans-neuronal propagation via extracellular vesicles such as exosomes, templating misfolding in healthy neurons and thereby facilitating disease progression. Using microfluidic devices, it has been demonstrated that exosomes mediate the trans-synaptic transfer of tau, a process enhanced by neuronal depolarization (Wang Y. et al., 2017). In an aging rhesus monkey model, abnormal tau phosphorylation in vulnerable neurons was linked to disrupted calcium homeostasis. Moreover, p-tau bound to microtubules can sequester APP-containing endosomes, potentially creating a feedforward mechanism that promotes Aβ production (Arnsten et al., 2021), as summarized in Figure 2.

Given the strong correlation between tau pathology and cognitive decline, tau has emerged as a pivotal therapeutic target in AD. *In vitro* studies have shown that methylene blue can inhibit the aggregation of both tau and Aβ (Maccioni et al., 2010). Furthermore, research in a novel mouse model of AD tangle pathology demonstrated that immunotherapy targeting pathological tau reduced the burden of harmful protein aggregates and prevented cognitive decline (Boutajangout et al., 2010), highlighting the promising potential of tau-targeted strategies. The expanding understanding of tau’s multifaceted roles, from cytoskeletal destabilization to trans-neuronal propagation, solidifies its position as a pivotal driver of AD pathogenesis, warranting continued investment in targeting this protein for disease-modifying therapies.

### 2.3 The neuroinflammation hypothesis

The neuroinflammatory hypothesis posits that Aβ plaques and NFTs are not merely pathological end products but also act as persistent inflammatory stimuli. They drive the transformation of microglia and astrocytes from homeostatic states toward pro-inflammatory phenotypes, thereby initiating a chronic neuroinflammatory process (Liu et al., 2025). Once established, this process can become self sustaining through the release of inflammatory cytokines such as IL-1β, which in turn promote further Aβ production and exacerbate tau phosphorylation, forming a vicious, self propagating cycle (Gan et al., 2022). As the resident immune cells of the central nervous system, microglia are pivotal for maintaining brain homeostasis by engaging in neural repair, proteostasis, and the clearance of abnormal protein aggregates (Linnartz et al., 2012). In AD, this homeostatic function is disrupted. Studies report a significant 20%–35% increase in microglial activation across multiple brain regions in AD patients, including the frontal, temporal, parietal, and occipital lobes, as well as the cingulate cortex (Molina-Martínez et al., 2020), establishing this as a prominent neuroinflammatory hallmark.

This chronic activation is triggered by Aβ deposits and neuronal damage, which shift microglia toward a pro-inflammatory phenotype. The response is dual-edged: activated microglia cluster around Aβ plaques and can participate in their clearance (Kitazawa et al., 2004). Conversely, Aβ itself can induce the formation and activation of the NLRP3 inflammasome within microglia, further aggravating Aβ aggregation and spreading (Calvo Alvarez et al., 2025). This creates a feed-forward loop of mutual reinforcement between pathology and inflammation. Triggering receptor expressed on myeloid cells 2 (TREM2) emerges as a central regulator in this process, modulating microglial responses to Aβ pathology. Research indicates that TREM2, upon binding Aβ, modulates microglial inflammatory responses, migration, and phagocytic clearance of Aβ deposits (Liu et al., 2020; Shi et al., 2025; Wei et al., 2024). Consequently, loss of TREM2 function significantly impairs Aβ-induced microglial signaling and compromises endogenous Aβ degradation. Individuals carrying the APOE (apolipoprotein E) ε4 allele face a significantly higher risk of developing AD, with an earlier age of onset (Di Battista et al., 2016). ApoE4 is thought to accelerate disease progression by disrupting the balance between Aβ deposition and clearance (Lima et al., 2020). Current efforts are therefore focused on developing strategies to modulate APOE function or mimic the protective effects of the APOE ε2 allele. Furthermore, glial fibrillary acidic protein, specifically expressed by reactive astrocytes, serves as an important biomarker for assessing neuroinflammatory levels (Takemura et al., 2002).

The therapeutic potential of targeting this inflammatory axis remains a subject of investigation. The efficacy of non-steroidal anti-inflammatory drugs (NSAIDs) in AD, for instance, is inconclusive. While research by Hoozemans et al. (2011) and Hirohata et al. (2008) suggested that systemic NSAID use might prevent or delay AD onset, clinical trials in diagnosed patients have not yielded consistent benefits. This discrepancy strongly suggests that NSAIDs are only effective during a preclinical or early prodromal stage, highlighting the critical importance of early diagnosis and timed intervention for anti-inflammatory strategies.

### 2.4 The cholinergic system damage hypothesis

The cholinergic hypothesis, one of the earliest and most substantiated theories of AD etiology, posits that the degeneration of cholinergic neurons in the basal forebrain, particularly within the nucleus basalis of Meynert, and the consequent decline in acetylcholine (ACh) levels are primary drivers of cognitive dysfunction in AD (Morelli et al., 2017; Rasool et al., 1986; Spencer et al., 2010). This system is fundamental to core cognitive processes such as learning, memory, and attention.

The integrity of cholinergic signaling depends on the precise synthesis and release of ACh. A reduction in these processes is not merely a correlate but a contributor to disease progression, capable of triggering neuronal apoptosis and cognitive impairment (Prado et al., 2002; Wetzel and Brown, 1983). A significant reduction in choline acetyltransferase (ChAT) activity in the hippocampus and cortex of AD patients, a finding now recognized as a hallmark of AD pathogenesis (Davies and Maloney, 1976). It is now understood that core AD pathologies, including Aβ deposition and tau hyperphosphorylation, engage in a vicious cycle with cholinergic damage, exacerbating it through the activation of apoptotic pathways, induction of oxidative stress, and aggravation of neuroinflammation (Suresh et al., 2022).

The ACh metabolic pathway is well defined, involving synthesis by ChAT, synaptic release, signal transduction via muscarinic and nicotinic receptors, and signal termination by acetylcholinesterase (Baig et al., 2018; Saw et al., 2021). In AD, the observed decrease in ChAT activity and ACh levels disrupts this cycle, providing the biochemical foundation for the cholinergic hypothesis.

The most compelling translational support for this hypothesis comes from the therapeutic efficacy of acetyl-cholinesterase inhibitors (ChEIs). By inhibiting AChE (acetylcholinesterase), these drugs augment cholinergic transmission and have become a mainstay for the symptomatic treatment of AD. Direct biochemical validation comes from clinical studies. For instance, a six month trial in patients with mild to moderate AD demonstrated that treatment with different ChEIs led to measurable, drug specific changes in cerebrospinal fluid AChE activity, directly correlating pharmacological intervention with target engagement in the central nervous system (Hwang et al., 2016). This provides robust mechanistic support for cholinergic intervention as a viable strategy in AD management. It is important to note that the cholinergic deficit itself can exacerbate Aβ and tau pathology, and its severity is modulated by overarching processes like neuroinflammation and oxidative stress, creating a deleterious feedback loop.

### 2.5 The oxidative stress hypothesis

In living cells, essential metabolic processes required for maintaining homeostasis inevitably generate reactive oxygen species (ROS) (Fasae et al., 2021). Under physiological conditions, low to moderate concentrations of ROS play crucial roles in various processes, including immune response, inflammation, signal transduction, synaptic plasticity, and memory formation (Kishida and Klann, 2007). The balanced production of ROS and reactive nitrogen species (RNS) is also vital for cellular signaling and defense against infections (Fasae et al., 2021; Sies, 2015). However, the central nervous system (CNS) is particularly vulnerable to oxidative stress. This susceptibility stems from its high oxygen consumption and the age related accumulation of metal ions that catalyze the overproduction of ROS and RNS (Chiurchiù et al., 2016; Fasae et al., 2021). Substantial evidence underscores the pivotal role of oxidative stress in the pathogenesis of AD (Praticò, 2008). Notably, oxidative damage is widespread even in the early stages of AD. Furthermore, the brains of AD patients exhibit significantly higher levels of oxidative stress compared to age matched healthy individuals, suggesting that AD is not merely a consequence of aging but involves specific factors that exacerbate this imbalance (Galasko and Montine, 2010). Among the implicated mechanisms, mitochondrial dysfunction is of particular importance due to its central role in energy metabolism and redox homeostasis. Research indicates that Aβ accumulates within the mitochondria of AD patients, where it impairs the activity of respiratory chain complexes (III and IV) and reduces the oxygen consumption rate (Caspersen et al., 2005). This Aβ accumulation can directly induce ROS production (e.g., by elevating intracellular H2O2 and lipid peroxides), thereby triggering cell death, neurodegeneration, and the recruitment and activation of glial cells, which in turn initiates local inflammatory responses (Yang et al., 2016). Concurrently, oxidative stress can prompt post-mitotic adult neurons to aberrantly re-enter the cell cycle, leading to attempted DNA replication in the absence of cell division. This abortive cycle ultimately results in neuronal apoptosis, aneuploidy, and DNA damage (Gupta et al., 2021; Rodriguez-Blanco et al., 2008). Additionally, free radicals can activate transcription factors such as NF-κB, thereby upregulating the expression of genes associated with neurodegeneration, including pro-inflammatory cytokines and vascular adhesion molecules (Abd El Aziz et al., 2021).

Given the central role of oxidative stress in AD, antioxidant strategies aimed at scavenging free radicals and inhibiting ROS generation represent a promising therapeutic avenue. Current research explores various antioxidant approaches not only to mitigate the toxic effects of ROS but also to enhance regenerative capacity in the adult brain. For instance, a study by C. Behl et al. (1994) demonstrated that catalase, an enzyme that degrades H2O2, can protect cells from Aβ-induced toxicity, providing experimental validation for the feasibility of antioxidant intervention. The damaging effects of ROS and RNS extend to promoting Aβ aggregation, accelerating tau phosphorylation, and activating neuroinflammatory pathways, thereby serving as a critical nexus interconnecting the core hypotheses of AD.

### 2.6 The metal ion hypothesis

Trace metal ions are essential for maintaining metal homeostasis in the brain under physiological conditions. Age-related dyshomeostasis of these metals is considered a causal factor in the pathogenesis of AD (Braidy et al., 2017). Imbalances in ions such as iron (Fe^2+^), copper (Cu^2+^), and zinc (Zn^2+^) are closely implicated in AD progression.

The interaction between metals and AD pathology is well-established. As early as 1994, it was discovered that Aβ can react with Zn^2+^ and Cu^2+^ to form amyloid deposits (Bush et al., 1994). Subsequent research using micro-particle induced X-ray emission confirmed significantly elevated levels of Zn^2+^, Fe^2+^, and Cu^2+^ in the core and periphery of senile plaques in the amygdala of AD patients compared to neuropil. Furthermore, zinc levels in the neuropil of AD patients were significantly higher than in controls, suggesting a link between abnormal metal accumulation and AD pathology (Lovell et al., 1998). These metal ions significantly modulate the misfolding and aggregation of Aβ into plaques (Lovell et al., 1998).

The specific mechanisms by which metals exacerbate pathology are multifaceted. Zinc and copper can influence the production of Aβ by modulating the γ-secretase processing of its precursor protein, APP-C99 (Gerber et al., 2017). Spectroscopic studies have shown that Cu^2+^ and Zn^2+^ accelerate the deposition of Aβ_40_ and Aβ_42_, while Fe^2+^ induces the formation of fibrous amyloid plaques at neutral pH (Ha et al., 2007). In physiological buffers, aluminum, iron, and zinc potently enhance the aggregation rate of Aβ_42_ (Mantyh et al., 1993). Beyond Aβ, iron can also interact with hyperphosphorylated tau protein, potentially facilitating the formation of NFTs in AD (Guo et al., 2013).

Systemic alterations in metal metabolism also play a role. A study of 116 AD patients and 89 healthy controls revealed decreased serum iron, ferritin, transferrin concentrations, and altered expression of iron transport genes in peripheral blood mononuclear cells in AD (Crespo et al., 2014). This systemic iron dysregulation is hypothesized to reflect cerebral iron homeostasis disruption, particularly impaired cellular iron export, leading to intracellular iron accumulation that may contribute to AD pathophysiology via increased oxidative damage (Crespo et al., 2014).

Given the pivotal role of metal ions, chelators have emerged as a potential therapeutic strategy. These compounds target and bind redox-active metals interacting with Aβ, rendering them redox-inactive and disrupting pathological processes (Budimir, 2011). Studies demonstrate that metal chelators like EDTA can reverse the binding of Cu^2+^ to histidine residues in senile plaques, inducing structural loosening of Aβ’s β-sheet conformation. This chelation induced structural change may offer a novel approach to dissolving amyloid deposits (Dong et al., 2003). Furthermore, chelators can specifically modulate levels of copper, zinc, and iron in the AD brain. Their benefits are multi-fold: they not only inhibit metal-mediated Aβ aggregation but also scavenge ROS, alleviate oxidative stress, modulate neuroinflammation, and improve synaptic function, thereby exerting broad neuroprotective effects (Mazur et al., 2024; Singh et al., 2024). Experimentally, Zn^2+^ and Cu^2+^ promote the formation of insoluble Aβ aggregates *in vitro*, which can be effectively dissolved by chelators. Compounds like EGTA, TPEN, and clioquinol have been shown to significantly increase the solubility of Aβ in post mortem AD brain tissues (Cherny et al., 1999). Beyond traditional chelators (e.g., D-penicillamine for Wilson’s disease, desferrioxamine for hemochromatosis), a new generation of compounds designed to attenuate aberrant metal protein interactions in pathological states is under active development (Ritchie et al., 2004; Sales et al., 2019).

### 2.7 The hypothesis of brain cholesterol deficiency

Emerging evidence suggests that decreased brain cholesterol levels may trigger a cascade of pathological events leading to dementia in AD patients. This concept gained traction when Greeve et al. (2000) first reported significantly reduced expression of the enzyme 24-dehydrocholesterol reductase (DHCR24) in the brains of AD patients, suggesting a link between DHCR24 downregulation and AD pathogenesis (Greeve et al., 2000; Iivonen et al., 2002). DHCR24 is a essential enzyme in the final step of cholesterol biosynthesis. Critically, diverse AD risk factors, including Aβ toxicity, aging, oxidative stress, and chronic inflammation, have been found to suppress DHCR24 expression, leading to impaired cholesterol synthesis in the brain (Bai et al., 2022; Dhana et al., 2018; Solfrizzi et al., 2011). This positions DHCR24 downregulation as a potential central node connecting various etiological factors to cholesterol dyshomeostasis in AD (Bai et al., 2022).

Further evidence indicates that reduced DHCR24 expression contributes to AD and other neurodegenerative pathologies through multiple mechanisms, such as promoting Aβ production, inducing neuronal and glial apoptosis, exacerbating tau hyperphosphorylation, impairing autophagy, and fostering neuroinflammation (Bai et al., 2022; Crameri et al., 2006; Najem et al., 2016; Rohanizadegan and Sacharow, 2018). Synthesizing these findings, Bai et al. (2022) proposed the Brain Cholesterol Deficiency Hypothesis of AD. This theory posits that various AD-related risk factors converge to disrupt cerebral cholesterol metabolism in both animal models and patients, resulting in cholesterol deficiency. This deficit, in turn, drives core pathological processes like Aβ aggregation and tau hyperphosphorylation, ultimately culminating in dementia (Bai et al., 2022). This hypothesis opens new avenues for basic research and provides a novel theoretical framework for developing clinical interventions.

### 2.8 The APOE hypothesis

APOE is a crucial protein that facilitates the transport and redistribution of cholesterol and other lipids among cells by serving as a ligand for low density lipoprotein receptors (Mahley, 1988). In the central nervous system, APOE plays vital roles in maintaining lipid homeostasis, promoting neuronal repair, preserving synaptic integrity, and aiding in toxin clearance (Mahley et al., 2006).

The APOE gene exists in three major alleles (ε2, ε3, ε4), with the APOE ε4 allele representing the most potent genetic risk factor for sporadic AD. The APOE4 protein isoform is strongly implicated in AD pathogenesis through multiple interconnected pathways. APOE4 directly influences Aβ pathology by impairing the clearance of Aβ peptides and promoting their aggregation into plaques. Concurrently, APOE4 exacerbates tau-mediated pathology, accelerating the hyperphosphorylation of tau and the formation of NFTs (Jervies et al., 2021). The complex interplay between APOE4, amyloid deposition, and tauopathy solidifies its central role in driving disease progression.

### 2.9 Other scientific hypotheses

In addition to the major hypotheses discussed above, the pathogenesis of AD involves several other interconnected pathways, including glutamate excitotoxicity, insulin resistance, mitochondrial dysfunction, and impaired autophagy (Ashleigh et al., 2023; Bordi et al., 2016; Iovino et al., 2020; Onaolapo and Onaolapo, 2021; Zhang et al., 2021).

Glutamate, the primary excitatory neurotransmitter in the CNS, is essential for synaptic transmission and plasticity (Bleich et al., 2003). However, its dysregulation can lead to excitotoxicity, a process implicated in various neurodegenerative diseases, including AD. In AD, impaired astrocytic reuptake of glutamate leads to its accumulation in the synaptic cleft. This overactivation of extrasynaptic NMDA receptors promotes neuronal damage and is closely linked to aberrant glial responses and neuroinflammation (Scott et al., 2011), forming a vicious cycle that exacerbates disease progression.

Mitochondria, as the primary organelles for energy production in eukaryotic cells, are critical for cellular metabolism (Liu B. H. et al., 2024). The Mitochondrial Cascade Hypothesis positions mitochondrial dysfunction as a core driver of AD pathogenesis. This hypothesis proposes that an individual’s baseline mitochondrial function and the rate of its age related decline fundamentally determine the trajectory of amyloid deposition, tau pathology, and ultimately, the progression of cognitive decline (Ashleigh et al., 2023).

Autophagy is a vital cellular clearance process that degrades and recycles damaged proteins and organelles to maintain homeostasis (Liu et al., 2023). In the context of AD, this degradative pathway becomes dysfunctional. Although studies, such as one by Bordi et al. (2016) analyzing neurons from the hippocampal CA1 region of AD patients, have observed an upregulation of genes related to autophagosome formation and some lysosomal components at early disease stages (Bordi et al., 2016), this likely represents an initial, compensatory response that ultimately fails. The accumulation of autophagic vesicles and inefficient clearance of Aβ and pathological tau indicate a profound failure in autophagic flux, which significantly contributes to AD pathology (Zhang et al., 2021).

## 3 The mechanism of ginseng macromolecule active components in the treatment of AD

Given the complex and multifactorial pathogenesis of AD, therapeutic strategies capable of simultaneously modulating multiple pathological pathways are highly desirable. In this context, the non-saponin macromolecular components of ginseng (GPS, GP, and GGP) have emerged as promising multitarget agents. This section critically evaluates their therapeutic potential, organizing the evidence around the core AD pathologies they address, from Aβ and tau to neuroinflammation and cellular survival. The multi-target mechanisms of these ginseng macromolecular active components are comprehensively summarized in Table 2.

**TABLE 2:** Summary of therapeutic evidence for ginseng macromolecules and formulas in AD.

Drug/compound	Mechanism	Molecular/biological target	Key findings	Clinical status	References
GP4	Inhibition of Aβ aggregation and mitophagy activation	Aβ_42_; PINK1/parkin	Reduced Aβ_42_ release in brain organoids; delayed paralysis in *C. elegans*	Preclinical	Zhang et al., 2023b
NFP	Anti-amyloidogenic and mitochondrial protection	Aβ plaque; mitochondrial respiratory chain	Improved mitochondrial defects in HT22 cells; reduced Aβ deposition in 5XFAD mice	Preclinical	Shin et al., 2021
GP	Activation of pro-survival pathways and tau modulation	PI3K/Akt signaling; Bcl-2/Bax ratio; p-tau	Improved memory in Morris water maze; reduced hippocampal Aβ_1–42_ and p-tau levels in rats	Preclinical	Li et al., 2016
GGP	Inhibition of neuroinflammation and anti-apoptosis	Notch signaling pathway; NOS activity	Protected SH-SY5Y cells against Aβ_25–35_ toxicity; improved cognition in APP/PS1 mice	Preclinical	Fang et al., 2023
SZJN	Endogenous neural regeneration	Neural stem cells; neurotrophic factors	Rescued neuronal death and triggered neurogenesis in AD-like mice	Clinical (CFDA)	Xiao et al., 2020

### 3.1 GPS: targeting Aβ pathology, neuroinflammation, and oxidative stress

Among ginseng macromolecules, GPS have been the most extensively studied for their multi-faceted neuroprotective effects, primarily by mitigating the core pathological triad of AD: Aβ aggregation, chronic neuroinflammation, and oxidative stress. While the cholinergic and oxidative stress hypotheses highlight important aspects of AD, the amyloid cascade remains a central focus. In this regard, GPS have shown significant efficacy in mitigating Aβ pathology in multiple experimental models.

#### 3.1.1 Structural heterogeneity and extraction

GPS are among the most abundant bioactive macromolecules in *Panax ginseng* (Wang et al., 2021). These natural polymers, formed by glycosidic linkages, exhibit molecular weights ranging from tens of thousands to millions of Daltons. Structurally, GPS are classified into neutral and acidic polysaccharides, primarily composed of galacturonic acid, galactose, rhamnose, and arabinose (Dai et al., 2025; Liu S. et al., 2024; Müller et al., 1989). This structural diversity, including variations in monosaccharide composition, glycosidic bonds, and chain conformation, is considered the molecular basis for their diverse pharmacological activities (Sun, 2011; Wang et al., 2021). The extraction of GPS typically employs hot water, alkaline solutions, or EDTA, often enhanced by ultrasound or microwave assistance (Wang et al., 2021; Zhao et al., 2019). Subsequent purification through ion-exchange and gel permeation chromatography is crucial for obtaining well-defined fractions (Qi et al., 2021; Zhang et al., 2023a).

#### 3.1.2 Multi-target mechanisms of action

**FIGURE 3:**
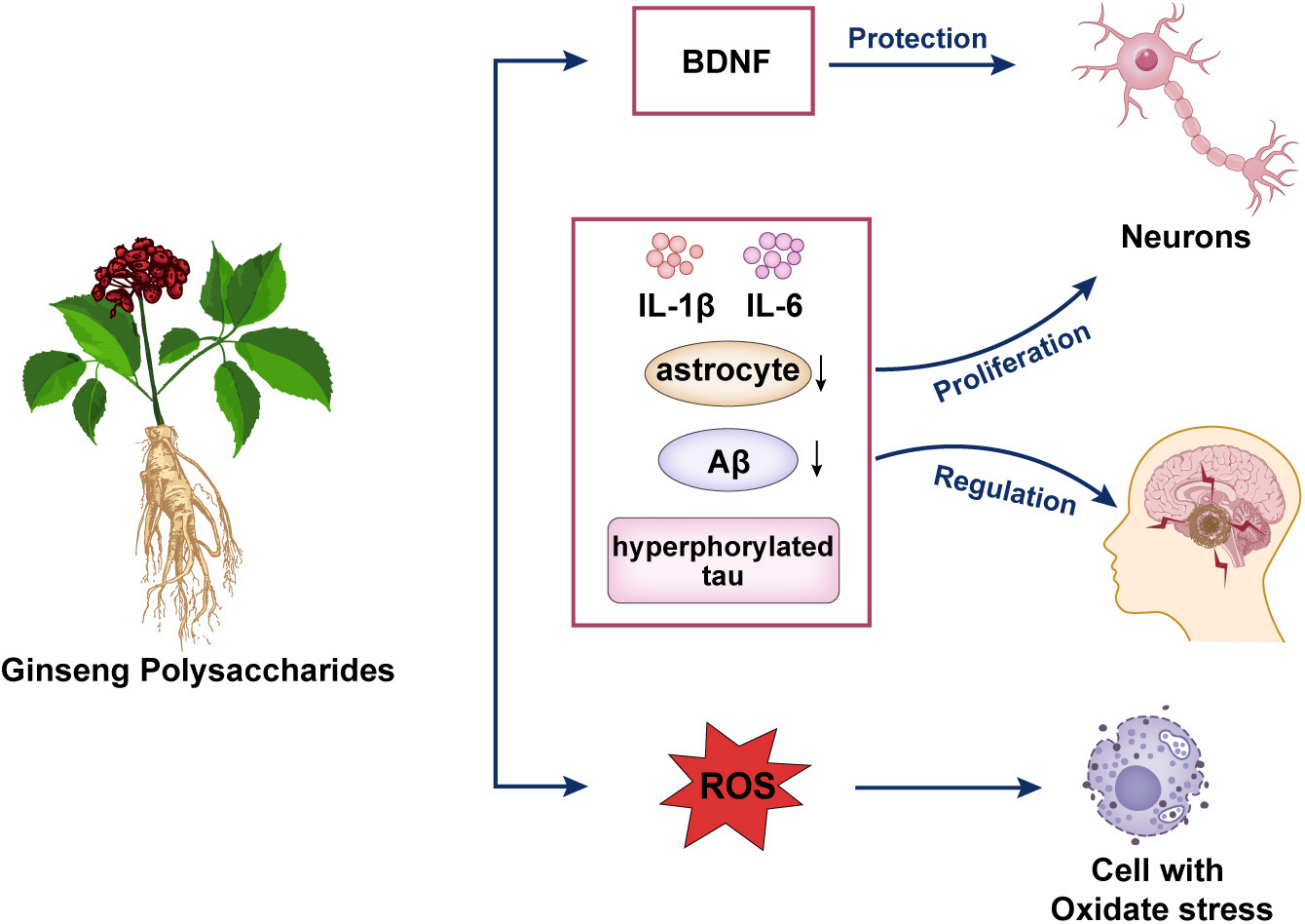
Multi-target neuroprotective mechanisms of GPS against AD.

GPS confer neuroprotection in AD through multi-target mechanisms, primarily involving the inhibition of Aβ pathology, attenuation of neuroinflammation, and mitigation of oxidative stress, as summarized in Figure 3.

Inhibition of Aβ aggregation and toxicity: A principal anti-AD mechanism of GPS is the direct interference with the amyloidogenic pathway. For instance, GP4, a 4.7 kDa polysaccharide, significantly suppresses Aβ aggregation in models including SH-SY5Y cells, brain organoids, and transgenic *C. elegans* (Zhang et al., 2023b). Similarly, the polysaccharide-rich non-saponin fraction (NFP) from Korean red ginseng inhibits Aβ deposition in 5XFAD transgenic mice (Shin et al., 2021).

Attenuation of neuroinflammation: GPS exhibit potent anti-neuroinflammatory properties. Water-soluble ginseng oligosaccharides ameliorate cognitive impairment by reducing hippocampal levels of pro-inflammatory cytokines (e.g., IL-1β, IL-6) and suppressing astrocyte activation (Jin et al., 2017). The broader anti-inflammatory activity of GPS is further highlighted by NFP, which inhibits microglial activation in AD models (Shin et al., 2021), and by GP4, which reduces Aβ-associated neuroinflammation (Zhang et al., 2023b).

Antioxidant and mitophagy-enhancing effects: The antioxidant capacity of GPS constitutes another vital mechanism. Studies have confirmed that GPS pretreatment can mitigate oxidative damage, such as that induced by ischemia-reperfusion (Yu et al., 2016; Zhao et al., 2014). Furthermore, GPS has been shown to protect red blood cells from oxidative stress by modulating glycolytic and gluconeogenic pathways (Liu et al., 2012; Wang et al., 2023). Beyond direct free radical scavenging, GPS also enhance mitochondrial quality control. NFP treatment improves mitochondrial function and neurogenesis in HT22 cells and 5XFAD mice (Xu et al., 2016). A study further revealed that NFP reduces tau protein accumulation and hyperphosphorylation in 3xTg AD mice, suggesting its therapeutic potential extends to tau pathology, potentially through mechanisms involving oxidative stress mitigation (Kim et al., 2024).

#### 3.1.3 Cross-species validation of GPS efficacy

The multi-target neuroprotective effects of GPS have been consistently validated across a broad spectrum of experimental models, underscoring their robust therapeutic potential and translational relevance.

Validation in cellular and organoid models: The core mechanisms of GPS were initially established in simplified *in vitro* systems. Studies in SH-SY5Y neuroblastoma and HT22 hippocampal neuronal cells confirmed the anti-Aβ, anti-inflammatory, and mitochondrial-protective effects of components like GP4 and NFP (Xu et al., 2016; Zhang et al., 2023b). The therapeutic relevance of GPS was further elevated in human-specific models, as demonstrated by GP4’s efficacy in reducing Aβ_42_ release and mitigating associated neuroinflammation in human AD brain organoids (Zhang et al., 2023b).

*C. elegans* models: In transgenic *C. elegans* models of AD, GP4 treatment significantly delayed Aβ_42_-induced paralysis, extended lifespan, and enhanced oxidative stress resistance, providing compelling whole-organism evidence for its neuroprotective benefits (Zhang et al., 2023b).

Efficacy in mouse models of AD: In transgenic mice such as 5XFAD and 3xTg models, GPS components like NFP alleviated major AD pathologies, including Aβ plaque load, tau hyperphosphorylation, neuroinflammation, and neuronal loss, which culminated in the amelioration of cognitive deficits (Kim et al., 2024; Shin et al., 2021).

This convergent evidence from reductionist *in vitro* systems, human-relevant organoids, invertebrate organisms, and mammalian rodent models solidifies the position of GPS as a promising and versatile candidate for the multi-target treatment of AD.

### 3.2 GP: modulating tau pathology and activating pro-survival pathways

**FIGURE 4:**
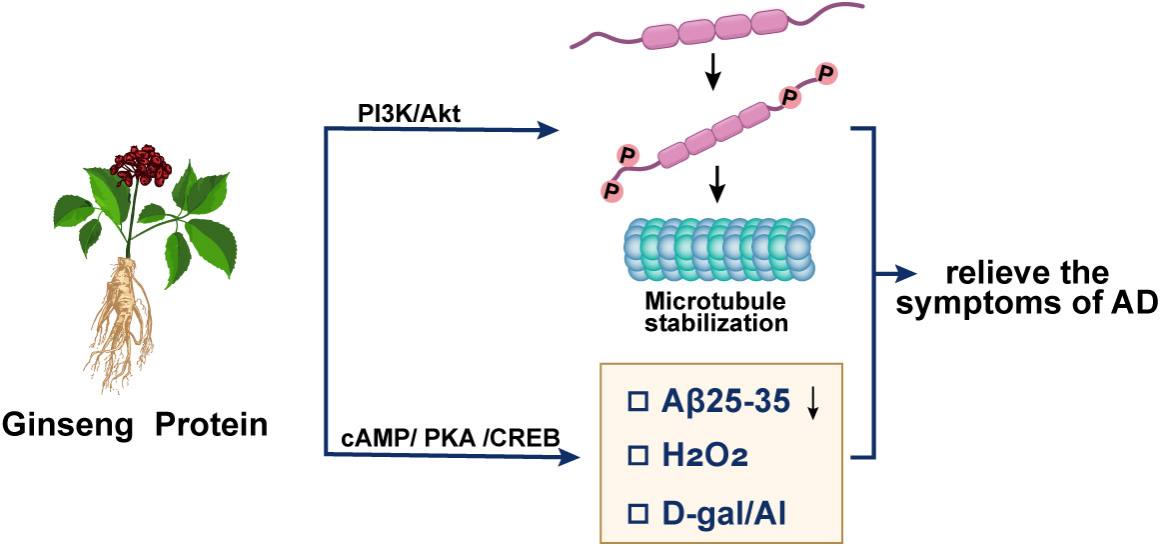
Therapeutic mechanisms of GP in promoting neuronal survival.

Complementing the anti-Aβ effects of GPS, GP appear to exert their primary neuroprotective influence primarily by modulating the other cornerstone of AD pathology: hyperphosphorylated tau, while concurrently activating critical pro-survival signaling cascades, as summarized in Figure 4.

#### 3.2.1 Proteomic landscape and extraction strategies

With advancements in proteomics, over 2,400 proteins have been identified in ginseng, involved in energy metabolism, stress response, and other physiological activities (Chen et al., 2017; Van Nguyen et al., 2021). The extraction of GP, which are thermally unstable, requires low-temperature methods. Combined strategies, such as the TCA/acetone-methanol/chloroform method, are recommended for comprehensive global protein extraction due to their ability to yield clearer electrophoresis patterns and more identifiable proteins (Van Nguyen et al., 2021).

#### 3.2.2 Core mechanisms: PI3K/Akt signaling and beyond

GP exerts its neuroprotective effects in AD primarily through the modulation of vital cell survival pathways and the attenuation of core pathologies.

Attenuation of tau hyperphosphorylation: A central mechanism is the inhibition of aberrant tau protein hyperphosphorylation. Research demonstrated that GP treatment in a D-galactose/AlCl_3_-induced AD rat model significantly reduced hippocampal levels of p-tau. This effect was mediated through the activation of the PI3K/Akt signaling pathway (Li et al., 2016). The ability of GP to suppress tau hyperphosphorylation directly contributes to the stabilization of microtubules and the maintenance of neuronal cytoskeletal integrity.

Activation of pro-survival pathways: Beyond its anti-tau effects, GP robustly activates central pro-survival signaling cascades. The same study identified that GP upregulates the PI3K/Akt pathway, leading to an increased Bcl-2/Bax ratio, a robust indicator of suppressed apoptotic activity, thereby promoting neuronal survival (Li et al., 2016). Furthermore, GP has been shown to inhibit AD-like pathophysiological changes by activating the cAMP/PKA/CREB pathway, a critical signaling axis for synaptic plasticity, learning, and memory (Li et al., 2017). The engagement of these complementary pathways underscores the multi-faceted neuroprotective capacity of GP.

#### 3.2.3 Evidence from rodent models of AD

The therapeutic potential of GP is compellingly evidenced in rodent models. In a chemical-induced AD rat model (D-galactose/AlCl_3_), GP administration dose-dependently improved spatial learning and memory in the Morris water maze test. Biochemically, GP treatment reduced hippocampal levels of both Aβ_1_–_42_ and p-tau, while concurrently activating the PI3K/Akt pathway and increasing the Bcl-2/Bax ratio (Li et al., 2016). This study provides a direct link between GP administration, the amelioration of core AD pathologies, the activation of pro-survival signals, and consequent cognitive improvement.

### 3.3 GGP: against cognitive decline via modulating neuroinflammation

**FIGURE 5:**
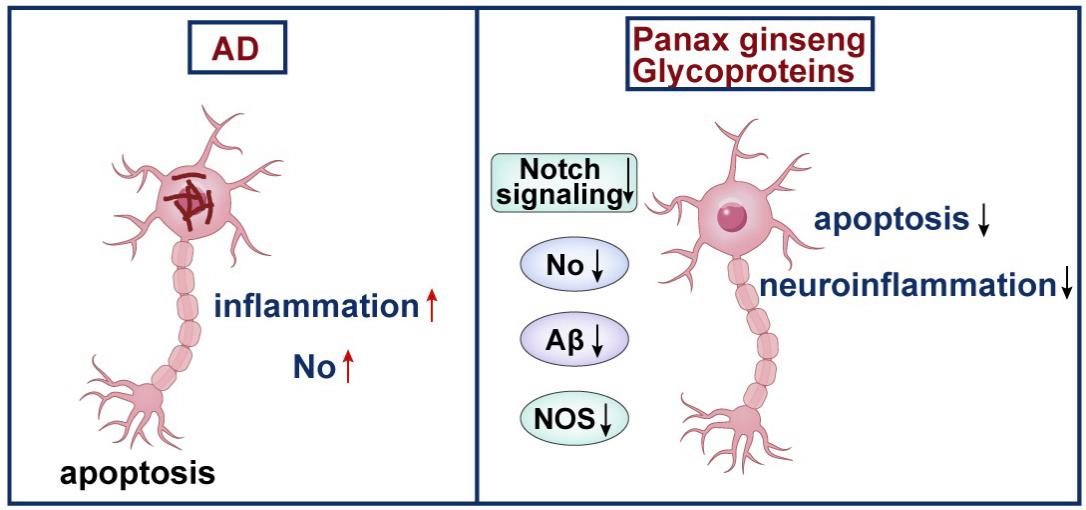
Neuroprotective actions of GGP via inhibition of neuroinflammation and apoptosis.

Beyond the direct targeting of Aβ and tau, the overarching process of neuroinflammation is a critical therapeutic target. GGP show potent activity in this domain, offering a unique approach to alleviating cognitive deficits in AD. The relevant functions of GGP are described in Figure 5.

#### 3.3.1 Unique glycoconjugate structure and purification

GGP are complexes formed by covalently bonded oligosaccharide chains and proteins, with O-glycosylation being a structural hallmark where the O-glycoside chain is α-linked to serine or threonine residues (Hu et al., 2022; Ye et al., 2016). The extraction of GGP, which integrates methods for both proteins and polysaccharides, typically involves water extraction from the ginseng core, followed by purification steps such as macroporous resin elution and ultrafiltration (Fang et al., 2023; Zhou et al., 2020).

#### 3.3.2 Mechanisms: Notch signaling inhibition and anti-apoptosis

GGP ameliorate cognitive deficits in AD through a multi-faceted mechanism that prominently involves the suppression of neuroinflammation and the prevention of neuronal death.

Improvement of learning and memory: The core therapeutic promise of GGP lies in its consistent ability to enhance cognitive functions. This is evidenced by significant improvements in behavioral tests, including the Morris water maze and nest-building test, across multiple AD models, from Aβ_25–35_-induced rats to APP/PS1 transgenic mice (Fang et al., 2023; Luo et al., 2018).

Inhibition of the neuroinflammatory Notch pathway: A principal molecular mechanism is the modulation of neuroinflammatory signaling. Luo et al. (2018) demonstrated that GGP improves cognitive impairment in AD model mice specifically by inhibiting the Notch signaling pathway, a critical regulator of glial activation and inflammatory response in the CNS (Bush et al., 1994; Fang et al., 2023).

Reduction of neuronal apoptosis: GGP confers direct neuroprotection by mitigating apoptotic cell death. The glycoprotein PGL-1 showed significant protection against Aβ_25–35_-induced apoptosis in SH-SY5Y cells, accompanied by an inhibition of S-phase cell cycle arrest and a reduction in intracellular NO levels and NOS activity (Luo et al., 2018).

#### 3.3.3 *In vivo* and *in vitro* neuroprotection

The neuroprotective mechanisms of GGP are substantiated by robust experimental evidence. *In vitro*, PGL-1 protected SH-SY5Y cells against Aβ_25–35_-induced toxicity (Luo et al., 2018). *In vivo*, GGP treatment improved cognitive function in both Aβ_25–35_-induced rats and APP/PS1 transgenic mice (Fang et al., 2023). A critical study confirmed that these improvements are linked to the inhibition of the Notch signaling pathway (Fang et al., 2023). Importantly, the biodistribution of GGP was confirmed by fluorescence imaging, showing their specific uptake by cerebral vascular endothelial cells and accumulation in the brain (Fang et al., 2023).

## 4 Clinical efficacy potential of active components of ginseng macromolecule

Ginseng has a long history of use in the context of AD treatment and is often combined with other traditional Chinese medicines in clinical practice. Compared to existing AD treatments, ginseng’s active components offer advantages such as mild side effects, good patient tolerance, and multi-target regulation. Currently, ginseng-containing Chinese herbal formulas have been developed for dementia treatment (Huang et al., 2021; Yang et al., 2019; Zhang et al., 2020). Preclinical studies primarily validate the neuroprotective effects and mechanisms of ginseng and its formulas through *in vitro* and animal models, providing experimental evidence for subsequent clinical translation.

### 4.1 Validation of the *in vitro* model

Organoids are three-dimensional cell cultures derived from stem cells that mimic the structure and function of native organs (Yi et al., 2021). In AD research, organoid models can simulate the three-dimensional structure of the brain, providing a platform that closely mimics the human physiological environment for studying AD’s pathological mechanisms and drug screening. Lee et al. (2022) established brain cerebral organoid models to investigate various neurodegenerative diseases. Zhang et al. (2024) also investigated the effects of semaglutide on AD using human AD-brain organoid models. These studies have demonstrated the effectiveness of organoid models in simulating the pathological process of AD.

Research on GPS demonstrates that GP4, a vital component, inhibits Aβ_42_ aggregation and neuroinflammation induced by Aftin-5, with effects validated in both SH-SY5Y cells and brain organoid models. Specifically, the study induced extracellular Aβ_42_ accumulation by treating cells with 100 μmol/L Aftin-5 for 18 h and established a 2-month-old brain organoid model. Immunofluorescence staining and enzyme-linked immunosorbent assorbent (ELISA) assays revealed that 24-h pre-treatment with GP4 significantly reduced Aβ_42_ release in the brain organoid culture medium, confirming its inhibitory effect on Aftin-5-induced Aβ aggregation (Zhang et al., 2023b).

In a cellular model, Shin et al. (2021) evaluated the effects of ginseng non-saponin polysaccharide components (NFP) on Aβ-mediated mitochondrial respiratory defects in HT22 mouse hippocampal neurons using hippocampal XFp metabolic analysis. The results demonstrated that NFP treatment improved mitochondrial defects in Aβ-treated HT22 cells.

Furthermore, in AD *in vitro* models, GP4 treatment can restore mitochondrial autophagy activity in neurons. Studies have shown that mitochondrial ATP production is crucial for neuronal survival, while mitochondrial dysfunction exists in AD models. After treating SH-SY5Y cells with GP4 and Aftin-5, it was found that high-dose GP4 pretreatment significantly increased oxygen consumption rate in cells, and both concentrations of GP4 elevated ATP levels, indicating its ability to protect cells from Aftin-5-induced mitochondrial dysregulation (Zhang et al., 2023b). Further research revealed that Aftin-5 treatment reduces the levels of crucial mitochondrial autophagy proteins PINK1 and parkin in human microbodies (MBs), while GP4 treatment significantly upregulates these two proteins. Immunofluorescence staining showed that GP4 enhances co-localization between mitochondria and lysosomes, accelerating mitochondrial degradation mediated by autophagy. In summary, GP4 can alleviate mitochondrial dysfunction in neurons of AD models by restoring mitochondrial autophagy activity (Zhang et al., 2023b).

### 4.2 Animal model studies

#### 4.2.1 *C. elegans* models

*C. elegans* models are classic invertebrate models for studying the molecular mechanisms of neurodegenerative diseases (Zhang et al., 2023b). Yang et al. (2020) previously used *C. elegans* (GMC101) to investigate the pharmacological effects of 6′′′-feruloylspinosin (6-FS) on Aβ. The GMC101 worm serves as an ideal model for replicating mammalian Aβ-related disease phenotypes, with its muscle cells continuously expressing Aβ_42_. Age-related paralysis and amyloid deposition in the body wall muscles only occur when the temperature rises from 20 °C to 25 °C. To investigate GP4’s capacity to modulate age-related Aβ_42_ toxicity and paralysis *in vivo*, Zhang et al. (2023b) treated GMC101 worms with GP4 at concentrations of 0.1, 1, or 10 mg/mL and analyzed paralysis phenotypes. The results demonstrated that 1 and 10 mg/mL GP4 effectively prevented Aβ_42_-induced paralysis, whereas the lower concentration of 0.1 mg/mL showed no beneficial effect. Further analysis revealed that early intervention with GP4 (from larval stage 4 to day 8) significantly reduced Aβ_42_-induced paralysis, extended GMC101 worms’ lifespan, and enhanced their tolerance to oxidative stress. Notably, GP4 could reverse Aβ_42_-induced cognitive impairment in CL2355 worms, a mechanism likely associated with inhibiting Aβ aggregation.

#### 4.2.2 Rat and mouse models

In rat models, Shin et al. (2021) investigated the effects of NFP on AD pathology in 5XFAD transgenic AD mouse models, including Aβ deposition, neuroinflammation, neurodegeneration, mitochondrial dysfunction, and adult hippocampal neurogenesis impairment. Additionally, the Y-maze test was used to evaluate NFP’s impact on Aβ-induced cognitive impairment in 5XFAD AD mouse models. Histological analysis demonstrated that NFP significantly reduced Aβ accumulation, neuroinflammation, neuronal loss, and mitochondrial dysfunction in the hypothalamus of AD 5XFAD mouse models.

Additionally, another study established an AD rat model through intraperitoneal injection of D-galactose (60 mg/kg/d) combined with intragastric injection of AlCl_3_ (40 mg/kg/d) over 90 days (Li et al., 2016). Starting from day 60, rats in the GP group received gavage of 0.05 or 0.1 g/kg of GP twice daily for 30 days. Behavioral performance was evaluated using the Morris water maze test, while hippocampal Aβ_1–42_ and p-tau levels were detected via ELISA. Real-time quantitative reverse transcription PCR and Western blot were employed to analyze mRNA and protein expressions of PI3K, Akt, p-Akt, Bcl-2, and Bax in the hippocampus. Results demonstrated that GP significantly improved memory function in AD rats, prolonged platform crossing time and quadrant occupancy percentage in the water maze test, reduced hippocampal Aβ_1–42_ and p-tau levels, and upregulated mRNA and protein expressions of PI3K, p-Akt/Akt, and Bcl-2/Bax. These findings indicate that GP exerts anti-AD effects by activating the PI3K/Akt signaling pathway (Li et al., 2016).

### 4.3 Clinical trials

Current clinical trials on ginseng and its active components for AD treatment primarily focus on ginseng extract applications, with no separate clinical trials conducted for ginseng protein/polysaccharide/glycoprotein extracts. Clinical trial results indicate that long-term ginseng extract intake significantly improves cognitive function and daily living abilities in AD patients, demonstrating high safety profiles (Lee et al., 2008).

In an open-label clinical trial, researchers randomly assigned consecutively enrolled AD patients to a ginseng group (58 cases, receiving 4.5 g of ginseng powder daily) or a control group (39 cases) (Lee et al., 2008). The treatment lasted 12 weeks, with cognitive performance monitored through the Mini-Mental State Examination (MMSE) and Alzheimer’s Disease Assessment Scale (ADAS) during therapy and 12 weeks post-treatment. No baseline differences were observed between the two groups in MMSE and ADAS scores. After 12 weeks of ginseng treatment, the ADAS cognitive subscale and MMSE scores showed significant improvement compared to baseline (*P* = 0.029 and *P* = 0.009), though these scores returned to control group levels after discontinuation (Lee et al., 2008). The study demonstrated that ginseng exhibited short-term clinical improvements in cognitive performance among AD patients.

In a separate study, Heo et al.’s (2012) randomly assigned 40 AD patients to three dosage groups (1.5 g/day, 3 g/day, and 4.5 g/day, with 10 cases in each) and a control group (10 cases), using the ADAS and MMSE to evaluate cognitive function over 24 weeks. Results showed that the high-dose group (4.5 g/day) demonstrated improvements in ADAS cognitive and non-cognitive subscales and MMSE scores as early as 12 weeks, demonstrating the potential therapeutic effects of heat-treated ginseng on cognitive function and behavioral symptoms in moderate-to-severe AD patients (Heo et al., 2012).

Although the above studies have confirmed the clinical value of ginseng extract, it is difficult to identify its core mechanism of action and active components due to the complex composition of the extract. Future clinical trials should focus on single active components such as GPS, proteins, and glycoproteins to provide more precise evidence for their clinical application.

### 4.4 Synergistic effects in herbal formulations

In the treatment of AD, ginseng and its active ingredients can be used alone or combined with other Chinese herbal medicines to enhance the therapeutic effect.

#### 4.4.1 Shenzao jiannao oral liquid (SZJN)

SZJN is a traditional China preparation, which was approved by the China Food and Drug Administration (CFDA) in 2002.

Through experiments including water maze tests, histopathological assessments, and protein level measurements, the research team discovered that SZJN, containing ginseng components, significantly improves cognitive dysfunction in AD model mice induced by Aβ_42_ and scopolamine (Xiao et al., 2020). It alleviates pathological damage, upregulates neurotrophic factor expression, and promotes endogenous neural regeneration.

*In vitro* studies demonstrated that SZJN, containing active ginseng components, significantly enhanced neural stem cell proliferation. The research team developed an AD cell model by transducing APP695swe gene into neural stem cells isolated from neonatal C57BL/6 mice hippocampus. This model indicates that SZJN may improve cognitive impairment by protecting neurons and triggering endogenous neural regeneration (Xiao et al., 2020), offering a potential therapeutic approach for AD.

#### 4.4.2 Fuzheng Quxie Decoction (FQD)

This study employed HPLC and LC-MS/MS to analyze the components of FQD, a traditional Chinese herbal compound for AD treatment (Yang et al., 2017). Three major components, ginsenoside Rg1, ginsenoside Re, and coix seed glycoside, were detected in the brains of mice treated with FQD, confirming its ability to cross the blood-brain barrier. In the SAMP8 mouse model of accelerated aging, FQD significantly improved learning and memory deficits in the Morris water maze test, as evidenced by shortened escape latency (*p* < 0.01), increased quadrant swimming time on the original platform (*p* < 0.05), and enhanced neuronal density and Nissl bodies in the hippocampal CA1 region. Molecular mechanism studies revealed that FQD reduces p-tau protein expression while upregulating protein phosphatase 2A (PP2A) and NMDAR subunit NR2A (*p* < 0.01). Its protective effects may involve inhibiting hyperphosphorylation of hippocampal tau protein through NMDAR/PP2A-related proteins (Yang et al., 2017).

#### 4.4.3 Huan Nao Yi Cong formula (HYD)

HYD has demonstrated remarkable neuroprotective effects in AD treatment. Studies indicate that HYD significantly improves cognitive function in AD model rats induced by Aβ_1–42_ by downregulating inflammatory factors (e.g., IL-1 and TNF-α) and Aβ levels, while regulating apoptosis-related proteins (e.g., Bcl-2, Bax, Caspase-3), thereby alleviating inflammatory responses and cellular apoptosis (Wang Q. et al., 2017).

#### 4.4.4 Others

Clinical trials led by Jia et al. (2018) demonstrated that Sailuotong, a compound formula combining Chinese and Western medicines with ginseng, can safely and effectively improve mild to moderate vascular dementia. Another study evaluated the effects of Memo^®^ (a marketed dietary supplement containing 750 mg freeze-dried royal jelly, 120 mg ginkgo leaf extract, and 150 mg ginseng extract) on cognitive function in patients with mild cognitive impairment. Results showed that Memo^®^ significantly improved patients’ MMSE scores, suggesting its potential to enhance cognitive decline and early-stage vascular dementia or AD in elderly individuals (Yakoot et al., 2013).

The aforementioned compound formulations demonstrated clear therapeutic efficacy and low adverse reaction rates in studies, indicating that ginsenosides, GPS, GP, and GGP play significant roles in the synergistic treatment of AD. However, most current research on the pharmacological basis of these compound drugs still focuses on ginsenosides, with insufficient exploration of the synergistic mechanisms of other active components, requiring further investigation.

## 5 Challenges and prospects

As the primary cause of dementia, AD has emerged as one of the most burdensome, lethal, and socioeconomically costly diseases in global public health this century. Its pathogenesis involves complex interactions across multiple dimensions and factors, which remain incompletely understood. While existing research has partially revealed correlations between primary pathological processes such as Aβ aggregation, tau protein hyperphosphorylation, and neuroinflammation, many core questions require further exploration (Shi et al., 2022). For instance: What is the source of free radicals in lipid bilayers that initiate single-electron oxidation of the S atom at Met residue 35 of Aβ_42_? Does the binding of Cu^2+^ to His cause electron release from the Tyr OH group at Aβ_42_ residue 10, leading to oxidative damage (Perluigi et al., 2024)? Further studies are needed to clarify whether microglial responses specifically target amyloid plaques, or also mediate toxicity induced by tau pathology or provide protection against tau (Scheltens et al., 2021). Elucidating these critical mechanistic questions will provide theoretical foundations for discovering new therapeutic targets.

The development of AD therapeutics has long grappled with high failure rates. Significant pathophysiological differences between animal models and human patients make it challenging to validate compounds effective in models for clinical trial efficacy and safety. Compounded by AD’s chronic progression, long-term follow-up clinical trials not only substantially increase R&D costs but also significantly prolong development cycles. Given the highly complex pathological mechanisms of AD, single-target drugs prove inadequate for effectively halting disease progression (Ogos et al., 2024). Future efforts should focus on developing multi-target strategies that simultaneously inhibit Aβ production and aggregation, regulate tau phosphorylation levels, and reduce neuroinflammatory responses. Furthermore, staged combination therapies tailored to pathological characteristics at different disease stages should be designed to enhance treatment precision and efficacy.

Against this backdrop, active components in ginseng such as GPS, GP, and GGP have demonstrated potential therapeutic value (Hu et al., 2022; Shi et al., 2022; Zhang et al., 2023b). Current research confirms that these components exert therapeutic effects through mechanisms including Aβ aggregation inhibition (Li et al., 2016; Shin et al., 2021) and neuroinflammation modulation (Fang et al., 2023; Xu et al., 2016), though their precise molecular mechanisms require further elucidation. For instance, additional studies are needed to determine whether GP4 can regulate gut microbiota for AD treatment, and to explore whether GP4-mediated ROS production inhibition could serve as a therapeutic switch to reverse mitochondrial dysfunction and restore mitochondrial activity, thereby preventing Aβ-associated pathology (Zhang et al., 2023b). The clarification of these mechanisms will lay the foundation for developing precision-targeted drugs based on ginseng components.

However, the transition from basic research to clinical translation of ginseng active components still faces multiple challenges. Firstly, clinical evidence remains relatively weak. Current studies predominantly rely on crude ginseng extracts, while large scale, multicenter randomized double blind clinical trials targeting individual components (such as polysaccharides, proteins, and glycoproteins) are severely lacking. The safety, optimal dosage, and synergistic efficacy with existing drugs require urgent validation. Secondly, production processes and standardization pose significant challenges. These components exhibit complex structures and high heterogeneity, with extraction and purification efficiency influenced by factors such as processing conditions, ginseng origin, growth years, and processing methods. This results in substantial variations in composition, purity, and bioactivity across batches, making quality homogeneity difficult to achieve. Thirdly, the structure-activity relationship remains unclear. Strategies combining chemical synthesis and structural modification are needed to optimize activity and bioavailability, while synergistic effects of multi-component combinations require systematic evaluation. Fourthly, currently extracted ginsenosides and glycoproteins often exist in mixed forms with unstable compositions and ratios, and principal active components remain incompletely identified. Therefore, advancing the development of large-molecule drugs in traditional Chinese medicine necessitates adopting new technologies to improve formulation processes, establish efficient drug delivery systems, comprehensively assess therapeutic advantages, and explore novel administration routes. For instance, in-depth research is needed on the stability and efficacy of novel drug delivery systems like liposomes in encapsulating natural macromolecules (e.g., glycoproteins and polysaccharides) (Hu et al., 2022).

To ensure the reproducibility and translatability of ginseng macromolecule research, we propose the following specific recommendations for preclinical standardization:

Chemical fingerprinting: Establishing standardized HPLC/LC-MS profiles for GPS and GGP is essential to manage structural heterogeneity and ensure batch-to-batch consistency (Fang et al., 2023).

Extraction protocols: Extraction temperatures and solvents should be harmonized; for instance, low-temperature methods are critical for GP to prevent the loss of bioactivity in thermally unstable proteins (Van Nguyen et al., 2021).

Pharmacokinetic characterization: Longitudinal imaging techniques, such as fluorescence tracking, should be implemented to quantify the blood-brain barrier permeability and cerebral accumulation of these large molecules (Fang et al., 2020).

Standardized bioassays: Future research should increasingly utilize validated human-brain organoid platforms alongside diverse rodent models to bridge the physiological gap in AD drug screening and better predict clinical outcomes (Zhang et al., 2023b).

In summary, developing effective AD prevention and treatment strategies requires a systematic and integrated approach that targets multiple pathogenic mechanisms. Research on ginseng’s active macromolecular components (GPS, GP, and GGP) offers new perspectives and resources for multi-target AD therapy. Future efforts focusing on in-depth mechanism analysis, clinical translation, and standardized production processes will facilitate the transition from experimental research to clinical application, providing AD patients with novel natural drug candidates.

## 6 Conclusion

The global incidence of AD continues to rise, with current treatments only providing limited symptom relief and unable to delay or halt disease progression. Research on AD’s pathogenesis reveals its highly complex mechanisms, involving interactions across multiple pathways and systems. This complexity poses significant challenges for drug development while simultaneously creating important opportunities for studying and applying non-saponin components such as GPS, GP, and GGP.

In summary, GPS, GP, and GGP demonstrate significant neuroprotective effects across multiple AD models, including cellular models, organoids, rodent models, and nematodes, by targeting core pathological processes such as Aβ aggregation, tau protein hyperphosphorylation, and neuroinflammation. Current preclinical evidence suggests their potential as multi-target intervention candidates for AD prevention and treatment. However, further high-quality randomized controlled trials are required to validate their clinical efficacy, safety, and applicable patient populations. This review establishes a mechanism-based theoretical framework and translational medicine pathway for developing innovative AD drugs derived from ginseng’s non-saponin components.
